# Supplementary material for: The fail-safe mechanism of post-transcriptional silencing of unspliced HAC1 mRNA
Source: eLife. 2016 Oct 1;5:e20069. doi: 10.7554/eLife.20069 (PMC5114014; doi:10.7554/eLife.20069)
Supplement: Supplementary file 1. — See key below. DOI: http://dx.doi.org/10.7554/eLife.20069.018 [file elife-20069-supp1.docx]

**Supplementary file 1. Yeast strains used in this study.** See key below.
